# Nutritional Risk in Older Adults with Rheumatoid Arthritis: Sex-Specific Patterns and Clinical Implications of the Prognostic Nutritional Index

**DOI:** 10.3390/nu18040673

**Published:** 2026-02-19

**Authors:** Joan M. Nolla, Lidia Valencia-Muntalà, Laura Berbel-Arcobé, Diego Benavent, Paola Vidal-Montal, Pol Maymó-Paituvi, Montserrat Roig-Kim, Martí Aguilar-Coll, Javier Narváez, Carmen Gómez-Vaquero

**Affiliations:** Department of Rheumatology, IDIBELL-Hospital Universitari de Bellvitge, University of Barcelona, 08907 Barcelona, Spainpvidal@bellvitgehospital.cat (P.V.-M.);

**Keywords:** rheumatoid arthritis, nutritional risk, Prognostic Nutritional Index, ageing, sex differences

## Abstract

Background/Objectives: Nutritional risk is increasingly recognized as a relevant but under-assessed dimension of rheumatoid arthritis (RA), particularly in older adults managed in outpatient settings. Simple nutritional indices such as the Prognostic Nutritional Index (PNI) may help identify individuals at increased nutritional risk beyond conventional disease activity measures. This study aimed to characterize nutritional risk in older adults with RA using the Prognostic Nutritional Index, explore sex-specific patterns, and identify clinical associations of PNI variability, with complementary analyses focusing on high nutritional risk. Methods: We conducted an observational cross-sectional study including 275 consecutive adults aged ≥50 years with RA attending routine follow-up at a tertiary rheumatology clinic. Nutritional risk was assessed using the PNI, calculated from serum albumin and total lymphocyte count, and analyzed primarily as a continuous variable and secondarily using established cut-off values. Clinical characteristics, inflammatory markers, body mass index, laboratory parameters, and patient-reported outcomes were recorded. Analyses were stratified by sex. Multivariable linear regression models were used to identify factors associated with PNI variability, and complementary logistic regression analyses were performed to explore factors independently associated with high nutritional risk (PNI < 40). Results: More than half of the cohort (53.3%) exhibited PNI values compatible with nutritional risk. Men showed lower PNI values than women, with a markedly greater prevalence of high nutritional risk (18.0% vs. 5.0%, *p* < 0.001). In multivariable linear regression analyses, higher C-reactive protein levels and increasing age were independently associated with lower PNI values, whereas sex was not an independent determinant of PNI. In multivariable logistic regression analyses, increasing age and male sex were independently associated with high nutritional risk. In multivariable linear regression models restricted to men, hemoglobin emerged as the principal independent correlate of PNI. In complementary logistic regression analyses focusing on high nutritional risk (PNI < 40), hemoglobin remained the sole independent predictor (OR = 0.94, 95% CI 0.91–0.98; *p* < 0.01), supporting a robust association with clinically relevant nutritional risk. Conclusions: Nutritional risk assessed by the PNI is common among older adults with RA. Although sex does not independently determine PNI as a continuous measure, male sex is associated with severe nutritional risk. The PNI captures a clinically relevant dimension of disease burden that extends beyond joint inflammation and traditional activity indices, supporting its use as a pragmatic nutritional screening tool in routine rheumatology practice.

## 1. Introduction

Rheumatoid arthritis (RA) is a chronic immune-mediated disease characterized by persistent joint and systemic inflammation [1]. This persistent inflammatory state accelerates biological ageing of the immune system and contributes to premature immune senescence [2].

Sustained immune-metabolic activation promotes a chronic low-grade inflammatory state, termed inflammaging, which drives oxidative stress, mitochondrial dysfunction and early cellular ageing [3]. In older adults, this inflammatory burden interacts with age-related metabolic changes and a progressive reduction in nutritional reserve, contributing to sarcopenia, metabolic dysfunction, anemia of chronic disease and loss of homeostatic reserve [4,5]. Together, these processes erode integrative physiological capacity in ways not captured by conventional disease activity indices and help explain the emergence of nutritional vulnerability in older adults with RA.

Nutritional status has growing relevance in RA because it reflects the intersection of inflammation, metabolic resilience and host immune competence. Understanding nutritional risk in RA is clinically important, as reduced nutritional reserve may affect treatment tolerance, increase susceptibility to infection and impair physical functioning, in line with robust evidence on disease-related malnutrition in chronic conditions [6]. Moreover, malnutrition appears to be highly prevalent among patients with RA and has been independently associated with increased all-cause mortality, thereby revealing an additional layer of disease burden beyond joint inflammation [7].

In clinical practice, comprehensive nutritional assessment is often complex and time-consuming, particularly in outpatient settings. In this context, the Prognostic Nutritional Index (PNI) [8], based exclusively on serum albumin and total lymphocyte count, provides a simple, pragmatic and readily accessible surrogate of nutritional risk. The PNI offers an interpretable estimate of protein reserve and systemic vulnerability and has demonstrated consistent prognostic value across a wide range of chronic diseases [9,10,11].

Nevertheless, evidence regarding its clinical role in RA remains limited and fragmented. Previous studies have mainly focused on specific outcomes such as disease activity [12], sarcopenia [13], infection risk [14] or mortality [15], rather than on the overall prevalence and clinical correlates of nutritional risk assessed by the PNI in well-characterized clinical cohorts. Consequently, data remain scarce on how frequently nutritional risk occurs in routine rheumatology practice and how PNI values relate to broader dimensions of disease burden beyond joint inflammation. Further studies are therefore needed to clarify the utility of the PNI for estimating nutritional risk, contextualizing disease impact and supporting patient stratification.

In chronic inflammatory diseases such as RA, nutritional risk should be understood as a multidimensional construct that extends beyond classical concepts of malnutrition. Laboratory-based indices such as the PNI incorporate components that are modulated by systemic inflammation and immune activation and therefore reflect an integrated state of nutritional reserve and physiological vulnerability rather than isolated nutritional impairment. In this context, the PNI may serve as a pragmatic marker of nutritional risk in the clinical sense, capturing disease-related systemic burden. This integrated view of nutritional risk also suggests that its clinical expression may vary across patient subgroups, including according to sex.

RA is also increasingly recognized as a disease characterized by relevant sex-related biological and clinical differences. Beyond prevalence, accumulating evidence indicates that immune responses, inflammatory burden, body mass index (BMI) and metabolic adaptations vary between men and women, highlighting the importance of a sex-informed perspective when interpreting clinical and biological indices in this disease [16,17,18].

In this context, we aimed to characterize the prevalence and clinical impact of nutritional risk in a cohort of older adults with RA, using the PNI as the primary analytic tool. We examined the distribution of PNI values, assessed their clinical and biochemical correlates, and identified factors independently associated with higher nutritional risk through multivariable modelling. This approach provides new insights into the burden of nutritional risk in RA and its potential relevance for patient stratification and clinical management.

## 2. Methods

### 2.1. Study Population

We conducted an observational cross-sectional study including 275 consecutive adults aged 50 years or older, of either sex, with RA attending routine follow-up visits at a tertiary university hospital rheumatology clinic. All participants fulfilled the 2010 ACR/EULAR classification criteria. The study was conducted within the framework of the Bellvitge Rheumatoid Arthritis–Life Impact and Comorbidity Evaluation cohort (BELL-RA-LIFE), a single-centre observational cohort specifically designed to include middle-aged and older adults with RA followed under routine clinical care. The cohort was conceived to characterize the global impact of the disease across ageing, with a particular focus on comorbidity burden, nutritional status, physical function, patient-reported outcomes, and health-related quality of life (HRQoL) in real-world settings.

Patients with conditions that could substantially confound the assessment of nutritional status or disease-related outcomes, including active malignancy, advanced heart or respiratory failure, chronic liver disease, or advanced chronic kidney disease, were excluded.

All participants provided written informed consent. The study was conducted in accordance with the Declaration of Helsinki and was approved by the Ethics Committee of Hospital Universitari de Bellvitge (protocol PR057/20, 28 October 2022).

### 2.2. Study Variables

#### 2.2.1. Sociodemographic Characteristics

Recorded sociodemographic variables included age, sex and body mass index (BMI). Lifestyle-related variables comprised smoking status (never, former, current) and self-reported physical activity, categorized as none, sporadic, regular low-intensity or regular high-intensity activity.

#### 2.2.2. Disease-Related Variables

- Clinical History and Serology

Key disease-related characteristics were documented, including disease duration, seropositivity for rheumatoid factor (RF) and anti-citrullinated peptide antibodies (ACPA), with their respective titers. Current pharmacological treatment was recorded and categorized as glucocorticoids, conventional synthetic disease-modifying antirheumatic drugs (csDMARDs), biologic DMARDs, or Janus kinase inhibitors.

- Disease Activity

Disease activity was assessed using the Disease Activity Score in 28 joints (DAS28) [19], a validated composite index calculated from tender and swollen joint counts, patient global assessment, and an acute-phase reactant (erythrocyte sedimentation rate [ESR]). In addition, systemic inflammation was evaluated using serum inflammatory markers, including ESR and C-reactive protein (CRP), which were also analyzed independently.

#### 2.2.3. Laboratory Parameters

Laboratory measurements included hemoglobin concentration, total lymphocyte count and serum albumin levels, obtained from routine blood analyses performed in proximity to study inclusion.

#### 2.2.4. Assessment of Nutritional Risk

The PNI was calculated as follows [8]:

PNI = (10 × serum albumin [g/dL]) + (0.005 × total lymphocyte count [per mm^3^]).

Lower values indicate poorer nutritional status. Commonly used interpretative thresholds were applied: (a) ≥45, adequate nutritional status; (b) 40–44.9, mild nutritional risk; and (c) <40, high nutritional risk.

#### 2.2.5. Physical Performance Measures

Handgrip strength was measured using a Jamar^®^ dynamometer in the dominant hand. Three attempts were performed, and the highest value was recorded. Low grip strength was defined using EWGSOP2 [20] sex- and age-specific thresholds: (a) <27 kg in men, (b) <16 kg in women. Gait speed was measured over a 4 m course at the participant’s usual walking pace. A gait speed < 0.8 m/s was classified as slow.

#### 2.2.6. Fatigue

Fatigue was assessed using the Functional Assessment of Chronic Illness Therapy-Fatigue (FACIT-F) scale [21]. Lower scores indicate greater fatigue; scores < 30 were considered indicative of clinically relevant fatigue.

#### 2.2.7. Health-Related Quality of Life

HRQol was evaluated using the 12-Item Short Form Survey (SF-12) [22]. Two summary measures were analyzed: (a) the Physical Component Score (PCS), reflecting physcal functioning and perceived physical health, and (b) the Mental Component Score (MCS), reflecting emotional well-being and mental health.

Higher scores indicate better perceived health status in both components.

### 2.3. Statistical Analysis

Descriptive statistics were used to summarize sociodemographic, clinical, laboratory and nutritional characteristics. Continuous variables were expressed as mean ± standard deviation (SD) or median (interquartile range, IQR) according to their distribution, assessed using the Shapiro–Wilk test. Categorical variables were presented as frequencies and percentages. Comparisons between men and women were performed using the chi-square test or Fisher’s exact test for categorical variables and Student’s t-test or Mann–Whitney U test for continuous variables, as appropriate.

The PNI was analyzed both as a continuous variable and as a categorical variable according to established cut-offs (adequate, mild risk, high risk). Given the observed differences in PNI between men and women, all analyses were stratified by sex.

Associations between PNI values and clinical or laboratory parameters were examined using Pearson or Spearman correlation coefficients, depending on data distribution.

To identify independent factors associated with PNI variability, all variables showing statistical significance in the univariate analyses were included in multivariable linear regression models, with PNI as the dependent variable. In addition, multivariable logistic regression analyses were performed to identify factors independently associated with high nutritional risk, defined as PNI < 40.

To incorporate a gender perspective, univariate and multivariable analyses were performed in the overall study population and stratified by sex, with separate models constructed for men and women.

All statistical tests were two-sided, and a *p*-value < 0.05 was considered statistically significant.

## 3. Results

Baseline sociodemographic and clinical characteristics of RA patients are summarized in Table 1. Within the cohort, 69.5% (n = 191) were women and 30.5% (n = 84) were men.

Men were older than women (71.9 ± 8.5 vs. 67.5 ± 8.8 years, *p* < 0.001) but had shorter disease duration (12.5 ± 9.6 vs. 16.8 ± 10.3 years, *p* < 0.01). No sex differences were observed in ESR, CRP, or autoantibody status. Women showed higher disease activity, reflected by higher DAS28 scores (2.9 ± 1.1 vs. 2.5 ± 1.2, *p* < 0.01), and poorer HRQol in both the physical and mental components of the SF-12.

In the overall series, according to PNI values, 53.3% of patients exhibited nutritional risk, which was mild in 44.5% and high in 8.8%.

PNI values were lower in men than in women (44.0 [40.0–46.0] vs. 45.0 [43.0–46.0], *p* < 0.05). The frequency of nutritional risk was higher in men compared with women (61.5% vs. 49.7%, *p* < 0.01), and a markedly higher proportion of men presented high nutritional risk (18.1% vs. 4.7%, *p* < 0.001).

A multivariate linear regression analysis was performed with the PNI as the dependent variable, including all variables with statistical significance in the univariate analyses. The final model explained a modest proportion of the variance in PNI (adjusted R^2^ = 0.106). Higher CRP levels were independently associated with lower PNI values (B = −0.069, 95% CI −0.110 to −0.029; *p* = 0.001). Age was also independently and inversely associated with PNI (B = −0.096, 95% CI −0.153 to −0.040; *p* = 0.001).

A multivariable logistic regression analysis was performed to identify factors independently associated with high nutritional risk, defined as PNI < 40. In the final model, increasing age was independently associated with a higher risk of severe nutritional impairment (OR 1.086, 95% CI 1.029–1.146; *p* = 0.003). After adjustment for age, male sex remained associated with high nutritional risk, with men showing higher likelihood of high nutritional risk compared with women (OR 3.42, 95% CI 1.39–8.40; *p* = 0.007).

In women, PNI was only associated with age, showing a weak inverse correlation (r = −0.169, *p* < 0.05).

In men, PNI showed multiple significant associations. It was inversely correlated with age (r = −0.339, *p* < 0.01), CRP (r = −0.287, *p* < 0.01), and DAS28 (r = −0.307, *p* < 0.01), and positively correlated with BMI (r = 0.267, *p* < 0.05) and hemoglobin levels (r = 0.389, *p* < 0.01). Progressive multivariable linear regression models identified hemoglobin as the principal independent factor associated with PNI variability (Table 2). In the first model, hemoglobin alone accounted for 15.2% of the variance in PNI (R^2^ = 0.152; adjusted R^2^ = 0.141), with a significant positive association (β = 0.389, *p* < 0.001). The addition of the SF-12 mental component score in Model 2 increased the explained variance to 20.3% (adjusted R^2^ = 0.182), with both hemoglobin (β = 0.364, *p* = 0.001) and mental health (β = 0.228, *p* = 0.029) remaining independently associated with PNI.

Model 3 showed that BMI contributed further to PNI variability, increasing R^2^ to 0.260 (adjusted R^2^ = 0.231). Hemoglobin (β = 0.333, *p* = 0.001), the SF-12 mental component score (β = 0.242, *p* = 0.017) and BMI (β = 0.241, *p* = 0.018) were all independently associated with PNI. In the final model (Model 4), CRP added a modest but significant negative contribution (β = −0.205, *p* = 0.040), increasing the explained variance to 30.0% (adjusted R^2^ = 0.263). Overall, in men, higher hemoglobin levels, better mental health status and higher BMI were independently associated with higher PNI values, whereas higher CRP levels were associated with lower PNI. The proportion of explained variance remained moderate.

A complementary logistic regression analysis was performed in men to explore factors independently associated with high nutritional risk (PNI < 40). Hemoglobin was the only variable that remained significantly associated, showing an inverse relationship with the odds of high nutritional risk (OR = 0.94, 95% CI 0.91–0.98; *p* < 0.01). Thus, lower hemoglobin levels were associated with a higher probability of high nutritional risk.

## 4. Discussion

In this cross-sectional study of predominantly older adults with RA, nutritional risk assessed by the PNI was common and showed apparent sex-related differences at the descriptive level. More than half of the cohort exhibited PNI values compatible with nutritional risk, with men displaying significantly lower PNI values, a higher frequency of nutritional risk and a markedly greater prevalence of high nutritional risk compared with women. Multivariable analyses demonstrated that age and inflammatory burden were the main independent determinants of PNI values, whereas sex was not independently associated with PNI when analyzed as a continuous variable after adjustment for age and inflammatory markers.

Before interpreting these associations, it is important to clarify how inflammatory burden was defined in the present study. In line with routine RA care, inflammatory burden was assessed using clinically integrated measures, including the DAS28 and acute-phase reactants (ESR and CRP). The DAS28 is a composite index that captures both systemic inflammation and clinical joint involvement and represents the standard tool for evaluating disease activity and guiding treatment decisions in treat-to-target strategies. Accordingly, the inflammatory burden in this study reflects clinically meaningful disease activity rather than detailed cytokine-level or cellular immune activation.

In RA, lower PNI values should be interpreted with caution. Given the strong influence of systemic inflammation and chronic disease burden on serum albumin and lymphocyte count, the PNI in this setting is best viewed as a marker of disease-related nutritional and physiological vulnerability rather than as an indicator of isolated nutritional impairment. This perspective supports a cautious, clinically oriented interpretation of the PNI as a screening tool for nutritional risk in chronic inflammatory disease.

In multivariable linear regression models, higher CRP levels and increasing age were independently associated with lower PNI values, confirming the central role of systemic inflammation and ageing-related processes in nutritional vulnerability among patients with RA. The final model explained a modest but clinically meaningful proportion of the variance in PNI, consistent with the multifactorial nature of nutritional risk in chronic inflammatory disease.

Although grip strength and gait speed are widely used markers of physical function and global vulnerability, their associations with PNI did not reach statistical significance in this cohort. This finding suggests that nutritional vulnerability, as captured by the PNI, may represent a dimension of disease-related vulnerability that is partially independent from functional performance. Alternatively, the lack of association may reflect the relatively preserved functional status of this ambulatory cohort and the multifactorial determinants of physical performance in RA.

Notably, although sex was not independently associated with PNI values across the full spectrum, a complementary multivariable logistic regression analysis revealed that male sex was independently associated with a higher likelihood of severe nutritional impairment, defined as PNI < 40 after adjustment for age. In this model, increasing age and male sex both emerged as significant independent predictors of high nutritional risk. This apparent discrepancy between linear and logistic models reflects the underlying distribution of PNI values, in which mean differences between sexes are modest, but men show a disproportionate accumulation of low PNI values at the lower tail of the distribution. These findings suggest that sex differences in nutritional vulnerability may become particularly relevant at the extreme end of the PNI distribution, rather than influencing average PNI values.

In women, despite slightly higher disease activity, high nutritional risk was uncommon and PNI showed only a weak association with age, with no meaningful relationships with inflammatory markers, disease activity or patient-reported outcomes. This pattern suggests a more gradual, age-related decline in nutritional reserve, largely independent of RA-specific clinical features. In contrast, in men, nutritional risk emerged as a clinically meaningful dimension of disease burden, associated with systemic inflammation, higher disease activity, lower BMI and poorer mental HRQol. In multivariable analyses, hemoglobin emerged as the principal independent factor associated with PNI variability, reflecting the close relationship between nutritional risk and systemic vulnerability in older men with RA.

Taken together, these results support the use of simple laboratory-based indices such as the PNI to capture dimensions of disease impact that are not adequately reflected by conventional measures of inflammatory activity alone. The distinction between determinants of PNI as a continuous variable and predictors of severe nutritional risk highlights the importance of considering different analytical approaches when evaluating nutritional vulnerability in RA.

The clinical context of RA has evolved substantially over recent decades. Treat-to-target strategies [23], earlier diagnosis, and the widespread use of biologic and targeted synthetic DMARDs have reduced severe inflammatory complications and hospitalizations [24]. Consequently, most patients are now managed in outpatient settings with satisfactory control of synovitis. This therapeutic progress has shifted attention toward less visible domains of disease impact, including functional decline, fatigue and nutritional vulnerability, which are incompletely captured by conventional disease activity indices and may persist despite low or moderate inflammatory activity [25].

In this ambulatory setting, tools capable of identifying patients at increased nutritional risk are particularly relevant. Our findings support the use of nutritional risk assessment in routine RA care not as a marker of acute illness or disease severity, but as an indicator of underlying vulnerability that may influence long-term outcomes and overall disease impact. Accordingly, the PNI may be considered a pragmatic screening tool to identify patients who could benefit from closer nutritional evaluation or targeted interventions, rather than a diagnostic instrument per se [26,27]. Notably, the identification of male sex as an independent predictor of high nutritional risk suggests that particular attention may be warranted in older men with RA, especially those of advanced age.

RA is increasingly recognized as a disease with relevant sex-specific biological and clinical differences [16]. In the present study, sex differences in nutritional risk were largely explained by age when PNI was analyzed as a continuous variable; however, male sex remained independently associated with severe nutritional impairment. These findings suggest that nutritional vulnerability in RA does not simply parallel inflammatory activity or patient-reported burden, but may reflect sex-related immune and metabolic trajectories that are insufficiently captured by conventional clinical assessment [14,28,29,30].

In this context, the strong association between hemoglobin levels and PNI is particularly informative. Across multivariable analyses, hemoglobin emerged as the factor most consistently associated with PNI variability and the only variable independently associated with high nutritional risk (PNI < 40) in both linear and logistic models in men, supporting the robustness of the finding. This association should be interpreted within a non-causal framework, as hemoglobin levels in this population are influenced by multiple disease-related and comorbidity-related factors. Anemia of chronic disease, a frequent consequence of chronic inflammation and ageing-related processes, reflects alterations in iron handling, erythropoiesis and metabolic efficiency [5,30]. Hemoglobin may therefore act as an integrative marker linking inflammatory burden, nutritional reserve and systemic vulnerability in older adults with RA, rather than a marker of isolated dietary insufficiency [31]. Accordingly, hemoglobin should be viewed as an indicator of overall systemic vulnerability rather than a direct determinant of nutritional risk.

In men, other variables, including BMI, CRP and the mental component of HRQol, showed weaker and less consistent independent associations with PNI. These factors likely capture complementary dimensions influencing nutritional reserve in chronic inflammatory disease—such as systemic inflammation, body composition and psychological well-being—suggesting a contextual rather than primary role in driving nutritional risk [32,33].

Beyond biological mechanisms, social and behavioural factors may also contribute to the observed sex-related patterns of nutritional vulnerability [34,35]. In older adults, nutritional status is shaped not only by inflammatory and metabolic processes but also by living conditions, dietary behaviours and social support. Older men, particularly those living alone, have been shown to exhibit poorer diet quality and lower engagement in nutritional self-care [34,35]. Although not directly assessed in the present study, these factors may partially modulate the nutritional risk captured by the PNI and should be considered when interpreting sex-specific differences. These considerations further emphasize the multifactorial nature of nutritional vulnerability in RA.

Several strengths of this study deserve emphasis. The analysis was conducted within a well-characterized, real-world cohort of older adults with RA, with comprehensive clinical, laboratory, functional and patient-reported data collected under routine care conditions. The use of multivariable linear and logistic regression models allowed a more nuanced characterization of nutritional vulnerability, distinguishing determinants of overall PNI levels from predictors of severe nutritional impairment.

Nevertheless, certain limitations should be acknowledged. The cross-sectional design precludes causal inference and limits conclusions regarding temporal relationships between nutritional risk and clinical outcomes. In addition, the interpretation of sex-related differences in nutritional risk requires caution. Although sex was not independently associated with PNI when analyzed as a continuous variable, it emerged as a relevant factor when severe nutritional risk was defined using a dichotomous threshold. This finding likely reflects differences in the distribution of PNI values, with a higher concentration of very low PNI values among men rather than a consistent difference across the entire range of PNI. The use of dichotomized outcomes may therefore emphasize differences at the extreme end of nutritional impairment, and results should be interpreted in this context. Although the PNI provides a pragmatic and accessible screening measure, it does not replace comprehensive nutritional assessment and does not capture all dimensions of malnutrition, including dietary intake, micronutrient status or body composition. Dietary patterns were not standardized or formally assessed, which should be considered when interpreting interindividual variability in PNI values. In addition, inflammatory burden was assessed using standard clinical and laboratory measures, and no cytokine profiling or immune cell phenotyping was performed. While this limits mechanistic interpretation, it reflects the pragmatic, real-world design of the study and enhances the clinical applicability of the findings. Finally, the single-centre nature of the cohort may limit generalizability, although it also ensures methodological consistency and uniform clinical assessment.

The present study did not include a comparator cohort without RA as its primary objective was to examine nutritional risk within the specific clinical context of RA. Future comparative or pilot studies including individuals without inflammatory joint disease may help further define the broader applicability and predictive performance of the PNI across different populations. Beyond RA, the PNI may also be of interest in other rheumatic diseases with joint involvement and varying degrees of systemic inflammation. Conditions characterized by low-grade inflammatory activity, such as osteoarthritis, as well as diseases with higher systemic inflammatory burden, such as psoriatic arthritis, may represent relevant settings in which nutritional vulnerability contributes to overall disease impact. Further studies are warranted to explore the clinical relevance of the PNI in these contexts.

## 5. Conclusions

Nutritional risk assessed by the PNI is common among older adults with RA. Age and inflammatory burden are the main independent determinants of PNI values, while male sex is independently associated with a higher likelihood of severe nutritional impairment. These findings support the integration of simple nutritional screening tools into routine RA care to improve risk stratification and promote a more holistic, patient-centred approach to disease management. Longitudinal studies are needed to determine whether trajectories of nutritional risk predict clinical outcomes and to clarify the role of targeted nutritional and multidisciplinary interventions in RA.

## Figures and Tables

**Table 1:** Characteristics of the 275 RA patients, stratified by sex into men (n = 84) and women (n = 191).

	Men	Women	*p*-Value
Age (years)	71.9 ± 8.5	67.5 ± 8.8	<0.001
BMI (kg/m^2^)	27.5 ± 3.5	27.9 ± 5.4	ns
Underweight (n, %)	1 (1.2%)	0	ns
Normal range (n, %)	18 (21.4%)	64 (33.5%)	-
Overweight (n, %)	49 (58.3%)	72 (37.5%)	<0.01
Obese (n, %)	16 (19.1%)	55 (29%)	-
Smoking			ns
Never (n, %)	72 (85.7%)	164 (87.2%)	
Former (n, %)	1 (1.2%)	2 (1.1%)	
Current (n, %)	11 (13.1%)	22 (11.7%)	
Physical activity			ns
None (n, %)	31 (37%)	94 (50.3%)	
Sporadic (n, %)	16 (19%)	36 (19.3%)	
Regular with low intensity (n, %)	33 (39.2%)	55 (29.4%)	
Regular with high intensity (n, %)	4 (4.8%)	2 (1.1%)	
Hemoglobin (g/dL)	14.2 ± 1.5	13.3 ± 1.2	<0.001
Serum albumin (g/L)	42.9 ± 4.7	44.3 ± 3.5	<0.01
Total lymphocyte count	2.1 ± 1.2	2.4 ± 1.0	ns
Disease duration (years)	12.5 ± 9.6	16.8 ± 10.3	<0.01
RF + (n, %)	50/83 (60.2%)	123/168 (73%)	ns
RF titer (UI/L)	175 ± 224	208 ± 415	ns
ACPA + (n, %)	52/83 (62.6%)	115/167 (69%)	ns
ACPA titer (U/L)	571 ± 1040	369 ± 672	ns
Current medication			
Glucocorticoids (n, %)	46 (54.7%)	89 (46.5%)	ns
cDMARDs (n, %)	73 (86.9%)	172 (90%)	ns
bDMARDs (n, %)	20 (23.8%)	68 (36%)	<0.001
Jak inhibitors (n, %)	2 (2.4%)	10 (5%)	ns
ESR (mm/h)	24.3 ± 26.2	24.8 ± 20.8	ns
CRP (mg/dL)	10.1 ± 18.3	5.2 ± 6.1	<0.05
DAS28	2.5 ± 1.2	2.9 ± 1.1	<0.01
Remission (n, %)	49 (58.3%)	77 (40.5%)	ns
LDA (n, %)	16 (19%)	47 (24.5%)	ns
MDA (n, %)	15 (17.9%)	61 (32%)	<0.01
HDA (n, %)	4 (4.8%)	6 (3%)	ns
SF-12			
Mental health	51.5 ± 10.0	45.1 ± 11.4	<0.001
Physical health	42.5 ± 9.6	36.8 ± 9.5	<0.001
Low grip strength (n, %)	36 (42.9%)	108 (56.8%)	<0.05
Low gait speed (n, %)	12 (14.3%)	50 (26.5%)	<0.05
PNI median, [IQR] (*)	44.0 [40.0–46.0]	45.0 [43.0–46.0]	
Adequate nutritional status (n, %)	32 (38.5%)	96 (50.3%)	<0.01
Mild nutritional risk (n, %)	36 (43.4%)	86 (45.0%)	<0.01
High nutritional risk (n, %)	15 (18.1%)	9 (4.7%)	

(*) PNI could be calculated in 83 men and 191 women due to one missing albumin count. BMI, body mass index; RF, rheumatoid factor; ACPA, anti-citrullinated peptides antibodies; ESR, erythrocyte sedimentation rate; CRP, C-reactive protein; cDMARDs, conventional disease-modifying antirheumatic drugs; bDMARDs; biological disease-modifying antirheumatic drugs; DAS28, Disease Activity Score 28; LDA, low disease activity; MDA, moderate disease activity; HDA: high disease activity; SF-12: Short Form Health Survey-12. IQR, interquartile range.

**Table 2:** Multivariable linear regression models identifying factors associated with the PNI in men with RA.

	Constant	Coefficient	*p*	Adjusted R^2^
Model 1				
Hemoglobin	25.013	0.389	<0.001	0.141
Model 2				
Hemoglobin	20.623	0.364	<0.01	0.182
SF12 MH		0.228	<0.05	
Model 3				
Hemoglobin	12.532	0.333	<0.01	0.231
SF12 MH		0.242	<0.05	
BMI		0.241	<0.05	
Model 4				
Hemoglobin	15.154	0.295	<0.05	0.263
SF12 MH		0.235	<0.05	
BMI		0.236	<0.05	
CRP		−0.205	<0.05	

## Data Availability

The datasets used and analyzed during the current study are available from the corresponding author upon reasonable request.
